# Low-Rank Deep Convolutional Neural Network for Multitask Learning

**DOI:** 10.1155/2019/7410701

**Published:** 2019-05-20

**Authors:** Fang Su, Hai-Yang Shang, Jing-Yan Wang

**Affiliations:** ^1^Shaanxi University of Science & Technology, Xi'an, Shaanxi Province 710021, China; ^2^Northwest University of Political Science and Law, Xi'an, Shaanxi Province 710063, China; ^3^New York University Abu Dhabi, Abu Dhabi, UAE

## Abstract

In this paper, we propose a novel multitask learning method based on the deep convolutional network. The proposed deep network has four convolutional layers, three max-pooling layers, and two parallel fully connected layers. To adjust the deep network to multitask learning problem, we propose to learn a low-rank deep network so that the relation among different tasks can be explored. We proposed to minimize the number of independent parameter rows of one fully connected layer to explore the relations among different tasks, which is measured by the nuclear norm of the parameter of one fully connected layer, and seek a low-rank parameter matrix. Meanwhile, we also propose to regularize another fully connected layer by sparsity penalty so that the useful features learned by the lower layers can be selected. The learning problem is solved by an iterative algorithm based on gradient descent and back-propagation algorithms. The proposed algorithm is evaluated over benchmark datasets of multiple face attribute prediction, multitask natural language processing, and joint economics index predictions. The evaluation results show the advantage of the low-rank deep CNN model over multitask problems.

## 1. Introduction

### 1.1. Backgrounds

In machine learning applications, multitask learning has been a popular topic [1–9]. It tries to solve multiple related machine learning problems simultaneously. The motive is that, for many situations, multiple tasks are closely related, and the prediction results of different tasks should be consistent. Accordingly, borrowing the prediction of other tasks to help the prediction of a given task is natural. For example, in the face attribute prediction problem, given an image, the prediction of female gender and wearing long hair is usually related [10–14]. Moreover, in the problem of natural language processing, it is also natural to leverage the problems of part-of-speech (POS) tagging and noun chuck prediction, since a word with a POS of a noun usually appears in a noun chunk [15–19]. Multitask learning aims to build a joint model for multiple tasks from the same input data.

In recent years, deep learning has been proven to be the most powerful data representation method [20–32]. Deep learning methods learn a neural network of multiple layers to extract the hierarchical patterns from the original data and provide high-level and abstractive features for the learning problems. For example, for the face-recognition problems, a deep learning model learns simple patterns by the low-level layers, such as lines, edges, circles, and squares. In the median-level layers, parts of faces are learned, such as eyes, noses, mouths, etc. In the high-level layers, patterns of entire faces of different users are obtained. With the deep learning model, we can explore the hidden but effective patterns from the original data directly with multiple layers, even without domain knowledge and hand-coded features. This is a critical advantage compared to traditional shallow learning paradigms models.

**Remark 1 .**

If shallow learning paradigm is applied in this case, the model structure will not be sufficient to extract complex hierarchical features. The users of these shallow learning models have to code all these complex hierarchical features manually in the feature extraction process, which is difficult and some times impossible.

**Remark 2 .**

If other nonneural networks learning models is used, such as spectral clustering, the hidden pattern of input data features cannot be directly explored. For example, spectral clustering treats each data point as a node in a graph and separates them by cutting the graph. However, it still needs a powerful data representation method to build the graph and cannot work itself well with a high-quality graph. Meanwhile, neural network models, especially deep neural network models, have the ability to represent the hidden patterns of input data points and build the high-quality graph accordingly. Thus, the nonneural network models and neural network models are complementary. Most recently, deep learning has been found very effective for multitask learning problems. For example, the following studies have discussed the usage of deep learning for multitask prediction.

- Zhang et al. [33] formulated a deep learning model constrained by multiple tasks, so that the early stopping can be applied to different tasks to obtain good learning convergence. Furthermore, different tasks regarding face images, including facial landmark detection, head pose estimation, and facial attribute detection are considered together by using a common deep convolutional neural network.
- Liu et al. [34] proposed a deep neural network learning method for multitask learning problems, especially for learning representations across multiple tasks. The proposed method can combine cross-task data, and also regularize the neural network to make it generalized to new tasks. It can be used for both multiple domain classification problems and information retrieval problems.
- Collobert and Weston [15] proposed a convolutional neural network for multitask learning problem in natural language processing applications. The targeted multiple tasks include POS tagging, noun chunk prediction, named entity recognition, etc. The proposed network is not only applied to multitask learning, but also applied to semisupervised learning, where only a part of the training set is labeled.
- Seltzer and Droppo [35] proposed to learn a deep neural network for multiple tasks which shares the same data representations. This model is used to the applications of acoustic models with a primary task and one or more additional tasks. The tasks include phone labeling, phone context prediction, and state context prediction.

However, the relation among different tasks is not explored explicitly. Although the deep neural model can learn effective high-level abstractive features, without explicitly exploring the relation of different tasks, different groups of level features may be used to different tasks. Thus, the deep features are separated for different tasks, and the relationships among different tasks are ignored during the learning process of the deep network. To solve this problem, we propose a novel deep learning method by regularizing the parameters of the neural network regarding multiple tasks by low-rank.

### 1.2. Our Contributions

The proposed deep neural network is composed of four convolutional layers, three max-pooling layers, and two parallel fully connected layers. The convolutional layers are used to extract useful patterns from the local region of the input data, and the max-pooling layers are used to reduce the size of the intermediate outputs of convolutional layers while keeping the significant responses. The last two fully connected layers are used to map the outputs of convolutional and max-pooling layers to the labels of multiple tasks.

The rows of the transformation matrices of the full connection layers are corresponding to the mapping of different tasks. We assume that the tasks under consideration are closely related; thus, the rows of the transformation matrices are not completely independent to each other; thus, we seek such a transformation matrix with a minimum number of independent rows. We use the rank of the transformation matrix to measure the number of the independent rows and measure it by the nuclear norm. During the learning process, we propose to minimize the nuclear norm of one fully connected layer's transformation matrix. Meanwhile, we also assume that, for a group of related tasks, only all the high-level features generated by the convolutional layers and max-pooling layers are useful. Thus, it is necessary to select useful features. To this end, we propose to seek sparse rows for the second fully connected layer. The sparsity of the second transformation matrix is measured by its *ℓ*_1_ norm, and we also minimize it in the learning process. Of course, we hope the predictions of the two fully connected layers could be low-rank and sparse simultaneously and also consistent with each other. Thus, we propose to minimize the squared *ℓ*_2_ norm distance between the prediction vectors of the two fully connected layers. Meanwhile, we also reduce the prediction error and the complexity of the filters of the convolutional layers measured by the squared *ℓ*_2_ norms. The objective function is the linear combination of these terms.

We developed an iterative algorithm to minimize the objective function. In each iteration, the transformation matrices and the filters are updated alternately. The transformation matrices are optimized by the gradient descent algorithm, and the filters are optimized by the back-propagation algorithms.

### 1.3. Paper Origination

The rest parts of this paper are organized as follows. In Section 2, we introduce the proposed method by modeling the problem as a minimization problem and develop an iterative algorithm to solve it. In Section 4, we conclude the paper.

## 2. Proposed Method

### 2.1. Problem Modeling

Suppose we have a set of *n* data points for the training process, denoted as {*x*_1_,…, *x*_*n*_}, where *x*_*i*_ is the *i*-th data point. *x*_*i*_ could be an image (presented as a matrix of pixels) or text (a sequence of embedding vectors of words). The problem of multitask learning is to predict the label vectors of *m* tasks. For *x*_*i*_, the label vector is denoted as **y**_*i*_=[**y**_*i*1_,…,**y**_*im*_]^T^ ∈ {1, −1}^*m*^, where **y**_*ij*_=1 if *x*_*i*_ is a positive sample for the *j*-th task, and **y**_*ij*_=−1, otherwise.

**Figure 1:**
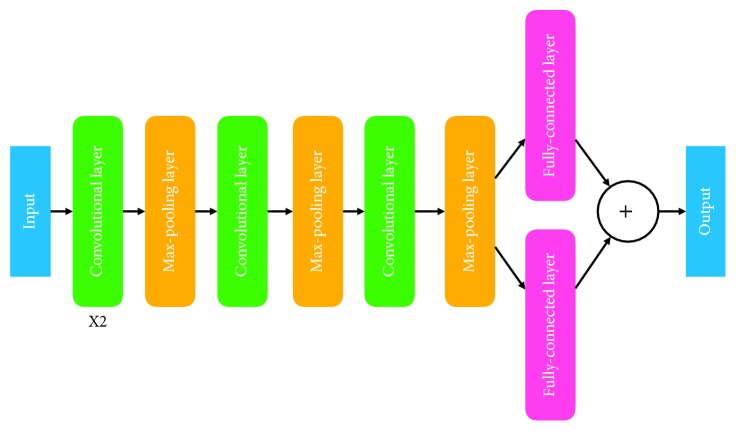
Sturcture of the proposed deep convolutional network.

To this end, we build a deep convolutional network to map the input data point to an output label vector. The network is composed of 4 convolutional layers, 3 max-pooling layers, and 2 parallel fully connected layers. The structure of the deep network is given in Figure 1. Please note that, for different types of input data, the convolutional and max-pooling layers are adjustable. For matrix inputs such as images, the layers perform 2D convolution and 2D max-pooling, while for sequences such as text, the layers conduct 1-D convolution and 1-D max-pooling.

We denote the intermediate output vector of the first 7 layers as *ϕ*(*x*) ∈ *R* ^*p*^, where *x* is the input, and *p* is the number of pools of the last max-pooling layer. The set of filters in the convolutional layers of *ϕ*(*x*) are denoted as Φ. The outputs of the two parallel fully connected layers are denoted as

(1) $$\begin{array}{c} f_{1}\left(x\right)=W_{1}\phi \left(x\right)\in R^{m},\\ f_{2}\left(x\right)=W_{2}\phi \left(x\right)\in R^{m}, \end{array}$$

where *W*_1_ ∈ *R* ^*m*×*p*^ and *W*_2_ ∈ *R* ^*m*×^ are the transformation matrix of the two layers. In the two fully connected layers map, the *p*-dimensional vector *ϕ*(*x*) of two vectors of *m* scores for *m* tasks. Each score measures the degree of the given data point belonging to the positive class. The two fully connected layers are corresponding to the low-rank and sparse prediction results of the network. By fusing their results, we can explore both the low-rank structure of the prediction scores of multitasks and also the sparse structure of the deep features learned from the network. In our model, the first fully connected layer **f**_1_(*x*) is responsible for the low-rank structure, while the second fully connected layer **f**_1_(*x*) is responsible for the sparse structure.

The final outputs of the network are the summation of the outputs of the two fully connected layers:

(2) $$\begin{array}{c} g_{1}\left(x\right)=W_{1}\phi \left(x\right)+W_{2}\phi \left(x\right)\in R^{m}. \end{array}$$

To learn the parameters of the deep network of **g**_1_(*x*), we consider the following four problems:

- *Low-Rank Regularization*. As we discussed earlier, the tasks are not completely independent from each other, but they are closely related to each other. To explore the relationships between different tasks, we learn a deep and shared representation *ϕ*(*x*) for the input data *x*. Based on this shared representation, we also request the transformation matrix *W*_1_ of one of the last fully connected layer to be of low-rank. The motive is that the *m* columns of *W*_1_ actually map the representation *ϕ*(*x*) to the *m* scores of *m* tasks. The rank of *W*_1_ measures the maximum number of linearly independent columns of *W*_1_. Thus, by minimizing the rank of *W*_1_, we can impose the mapping functions of different tasks to be dependent on each other and minimize the number of independent tasks. To measure the rank the matrix *W*_1_, rank(*W*_1_), we use the nuclear norm of *W*_1_, denoted as ‖*W*_1_‖_*∗*_. ‖*W*_1_‖_*∗*_ is calculated as the summation of its singular values:

(3) $$\begin{array}{c} {\left\|W_{1}\right\|}_{*}=\underset{l}{\sum }\varrho_{l}, \end{array}$$

  where *ϱ*_*l*_ is its *l*-th singular value. We propose to learn *W*_1_ by regularizing its rank as follows:

(4) $$\begin{array}{c} \underset{W_{1}}{\min}{\left\|W_{1}\right\|}_{*}. \end{array}$$

(ii) *Sparse Regularization*. We further regularize the mapping transformation matrix of the second fully connected layer by sparsity. The motive of the sparsity is that the effective deep features for different tasks might be different, and for each task, not all the features are needed. Although we learn a group of deep features in *ϕ*(*x*) and share it with all the tasks, for a specific task and its relevant tasks, only a small number of deep features are necessary, and feature selection is a critical step. For the purpose of features selection, we impose the sparsity penalty to the transformation matrix of the second fully connected layer, *W*_2_, since it maps the deep features to the prediction scores of *m* tasks. To measure the sparsity of *W*_2_, we use the *ℓ*_1_ norm of *W*_2_, which is the summation of the absolute values of all the elements of the matrix:

(5) $$\begin{array}{c} {\left\|W_{2}\right\|}_{1}=\underset{jk}{\sum }\left|{\left[W_{2}\right]}_{jk}\right|. \end{array}$$

  We minimize the *ℓ*_1_ norm of *W*_2_ to learn a sparse *W*_2_

(6) $$\begin{array}{c} \underset{W_{2}}{\min}\frac{1}{2}{\left\|W_{2}\right\|}_{1}. \end{array}$$

(iii) *Prediction Consistency*. The outputs of the two fully connected layers of low-rank and sparsity may give different results. However, they can be consistent with each other so that the prediction results can be low-rank and sparse simultaneously. To this end, we impose to minimize the squared *ℓ*_2_ norm distance between the prediction results of the two layers over all the training data points:

(7) $$\begin{array}{c} \underset{\Phi ,W_{1},W_{2}}{\min}\frac{1}{2}\overset{n}{\underset{i=1}{\sum }}\left\|W_{1}\phi \left(x_{i}\right)\right.-{\left.W_{2}\phi \left(x_{i}\right)\right\|}_{2}^{2}. \end{array}$$

(iv) *Prediction Error Minimization*. We also propose to learn an effective multitask predictor by minimizing the prediction error. To measure the prediction error of a data point, *x*, we calculate the squared *ℓ*_2_ norm distance between its prediction result *g*(*x*) and its true label vector **y**:

(8) $$\begin{array}{c} {\left\|y-g\left(x\right)\right\|}_{2}^{2}={\left\|y-\left(W_{1}\phi \left(x\right)+W_{2}\phi \left(x\right)\right)\right\|}_{2}^{2}. \end{array}$$

  We learn the parameters of the deep network by minimizing the errors over all the training data points:

(9) $$\begin{array}{c} \underset{\Phi ,W_{1},W_{2}}{\min}\frac{1}{2}\overset{n}{\underset{i=1}{\sum }}{\left\|y_{i}-\left(W_{1}\phi \left(x_{i}\right)+W_{2}\phi \left(x_{i}\right)\right)\right\|}_{2}^{2}. \end{array}$$

(v) *Complexity Reduction*. Finally, we regularize the filters of the convolutional layers, Φ, by the squared *ℓ*_2_ norms to prevent the network from being over complex:

(10) $$\begin{array}{c} \underset{\Phi }{\min}\frac{1}{2}{\left\|\Phi \right\|}_{2}^{2}. \end{array}$$

  The overall optimization problem is the weighted combination of the problems above:

(11) $$\begin{array}{c} \underset{\Phi ,W_{1},W_{2}}{\min}\left\{g=\frac{1}{2}{\left\|\Phi \right\|}_{2}^{2}+\frac{C_{1}}{2}\overset{n}{\underset{i=1}{\sum }}{\left\|y_{i}-\left(W_{1}\phi \left(x_{i}\right)+W_{2}\phi \left(x_{i}\right)\right)\right\|}_{2}^{2}+C_{2}{\left\|W_{1}\right\|}_{*}+\frac{C_{3}}{2}{\left\|W_{2}\right\|}_{1}+\frac{C_{4}}{2}\overset{n}{\underset{i=1}{\sum }}{\left\|W_{1}\phi \left(x_{i}\right)-W_{2}\phi \left(x_{i}\right)\right\|}_{2}^{2}\right\}, \end{array}$$

  where *C*_1_, *C*_2_, *C*_3_, and *C*_4_ are the weights of different objective terms, and *g* is the overall objective function. By optimizing this problem, we can obtain a deep convolutional network with a low-rank and sparse deep features for the problem of multitask learning.

### 2.2. Optimization

To solve the problem in (12), we use the alternate optimization method. The parameters are updated iteratively in an iterative algorithm. When one parameter is updated, others are fixed. In the following sections, we will discuss how to solve them separately.

#### 2.2.1. Updating *W*_1_

When we update *W*_1_, we fix *W*_2_ and Φ, remove the terms irrelevant to *W*_1_ from (12), and obtain the following optimization problem:

(12) $$\begin{array}{c} \underset{W_{1}}{\min}\left\{g_{1}\left(W_{1}\right)=\frac{C_{1}}{2}\overset{n}{\underset{i=1}{\sum }}{\left\|y_{i}-\left(W_{1}\phi \left(x_{i}\right)+W_{2}\phi \left(x_{i}\right)\right)\right\|}_{2}^{2}+C_{2}{\left\|W_{1}\right\|}_{*}+\frac{C_{4}}{2}\overset{n}{\underset{i=1}{\sum }}{\left\|W_{1}\phi \left(x_{i}\right)-W_{2}\phi \left(x_{i}\right)\right\|}_{2}^{2}\right\}, \end{array}$$

where *g*_1_ is the objective function of this problem. To solve this problem, we use the gradient descent algorithm. *W*_1_ is descended to the direction of the gradient of *g*_1_(*W*_1_):

(13) $$\begin{array}{c} W_{1}⟵W_{1}-\varsigma \nabla g_{1}\left(W_{1}\right), \end{array}$$

where ∇*g*_1_(*W*_1_) is the gradient function of *g*_1_(*W*_1_), and *ς* is the descent step size. To calculate the gradient function ∇*g*_1_(*W*_1_), we first split the objective into two terms:

(14) $$\begin{array}{c} g_{1}\left(W_{1}\right)=g_{11}\left(W_{1}\right)+g_{12}\left(W_{1}\right), \end{array}$$

where

(15) $$\begin{array}{c} g_{11}\left(W_{1}\right)=\frac{C_{1}}{2}\overset{n}{\underset{i=1}{\sum }}{\left\|y_{i}-\left(W_{1}\phi \left(x_{i}\right)+W_{2}\phi \left(x_{i}\right)\right)\right\|}_{2}^{2}+\frac{C_{4}}{2}\overset{n}{\underset{i=1}{\sum }}{\left\|W_{1}\phi \left(x_{i}\right)-W_{2}\phi \left(x_{i}\right)\right\|}_{2}^{2},\\ g_{12}\left(W_{1}\right)=C_{2}{\left\|W_{1}\right\|}_{*}. \end{array}$$

The first term *g*_11_(*W*_1_) is a quadratic term while *g*_12_(*W*_1_) is a unclear term. Thus, the gradient function of *g*_1_(*W*_1_) is the sum of the gradient functions of the two terms:

(16) $$\begin{array}{c} \nabla g_{1}\left(W_{1}\right)=\nabla g_{11}\left(W_{1}\right)+\nabla g_{12}\left(W_{1}\right), \end{array}$$

where ∇*g*_11_(*W*_1_) can be easily obtained as

(17) $$\begin{array}{c} g_{11}\left(W_{1}\right)=-C_{1}\overset{n}{\underset{i=1}{\sum }}\left(y_{i}-\left(W_{1}\phi \left(x_{i}\right)+W_{2}\phi \left(x_{i}\right)\right)\right)\phi {\left(x_{i}\right)}^{\text{T}}+C_{4}\overset{n}{\underset{i=1}{\sum }}\left(W_{1}\phi \left(x_{i}\right)-W_{2}\phi \left(x_{i}\right)\right)\phi {\left(x_{i}\right)}^{\text{T}}. \end{array}$$

To obtain the gradient function of *g*_12_(*W*_1_)=*C*_2_‖*W*_1_‖_*∗*_, we first decompose *W*_1_ by singular value decomposition (SVD):

(18) $$\begin{array}{c} W_{1}=U\sum V, \end{array}$$

where *U* and *V* are the two orthogonal matrices, ∑ is a diagonal matrix containing all the singular values. According to the Proposition 1 of [36], the gradient of ‖*W*_1_‖_*∗*_=*U*∑^−1^|∑|*V*; thus,

(19) $$\begin{array}{c} g_{12}\left(W_{1}\right)=C_{2}U\sum^{-1}\left|\sum \right|V. \end{array}$$

#### 2.2.2. Updating *W*_2_

To update *W*_2_, we also fix other parameters and remove the irrelevant terms:

(20) $$\begin{array}{c} g_{2}\left(W_{2}\right)=\frac{C_{1}}{2}\overset{n}{\underset{i=1}{\sum }}{\left\|y_{i}-\left(W_{1}\phi \left(x_{i}\right)+W_{2}\phi \left(x_{i}\right)\right)\right\|}_{2}^{2}+\frac{C_{3}}{2}{\left\|W_{2}\right\|}_{1}+\frac{C_{4}}{2}\overset{n}{\underset{i=1}{\sum }}{\left\|W_{1}\phi \left(x_{i}\right)-W_{2}\phi \left(x_{i}\right)\right\|}_{2}^{2}=g_{21}\left(W_{2}\right)+g_{22}\left(W_{2}\right), \end{array}$$

where

(21) $$\begin{array}{c} g_{21}\left(W_{2}\right)=\frac{C_{1}}{2}\overset{n}{\underset{i=1}{\sum }}{\left\|y_{i}-\left(W_{1}\phi \left(x_{i}\right)+W_{2}\phi \left(x_{i}\right)\right)\right\|}_{2}^{2}+\frac{C_{4}}{2}\overset{n}{\underset{i=1}{\sum }}\left\|W_{1}\phi \left(x_{i}\right)\right.-W_{2}\phi {\left.\left(x_{i}\right)\right\|}_{2}^{2}, \end{array}$$

is a quadratic term, and

(22) $$\begin{array}{c} g_{22}\left(W_{2}\right)=\frac{C_{3}}{2}{\left\|W_{2}\right\|}_{1}, \end{array}$$

is a *ℓ*_1_ norm term. We also use the gradient descent algorithm to update *W*_2_:

(23) $$\begin{array}{c} W_{2}⟵W_{2}-\varsigma \nabla g_{2}\left(W_{2}\right), \end{array}$$

where

(24) $$\begin{array}{c} \nabla g_{2}\left(W_{2}\right)=\nabla g_{21}\left(W_{2}\right)+\nabla g_{21}\left(W_{2}\right),\\ \nabla g_{21}\left(W_{2}\right)=-C_{1}\overset{n}{\underset{i=1}{\sum }}\left(y_{i}-\left(W_{1}\phi \left(x_{i}\right)+W_{2}\phi \left(x_{i}\right)\right)\right)\phi {\left(x_{i}\right)}^{\text{T}}-C_{4}\overset{n}{\underset{i=1}{\sum }}\left(W_{1}\phi \left(x_{i}\right)-W_{2}\phi \left(x_{i}\right)\right)\phi {\left(x_{i}\right)}^{\text{T}}. \end{array}$$

To obtain the gradient function of *g*_21_(*W*_2_), we rewrite *W*_2_ and ‖*W*_2_‖_1_ as follows:

(25) $$\begin{array}{c} W_{2}=\left[\begin{array}{c} w_{21}\\ \vdots \\ w_{2m} \end{array}\right], \end{array}$$

where

(26) $$\begin{array}{c} {\left\|W_{2}\right\|}_{1}=\overset{m}{\underset{i=1}{\sum }}{\left\|w_{2i}\right\|}_{1}=\overset{m}{\underset{i=1}{\sum }}{\left\|w_{2i}\right\|}_{1},\\ {\left\|w_{2i}\right\|}_{1}=\overset{d}{\underset{j=1}{\sum }}\left|w_{2ij}\right|=\overset{d}{\underset{j=1}{\sum }}\frac{w_{2ij}^{2}}{\left|w_{2ij}\right|}=w_{2i}\;\;\mathrm{diag}{\left(\left|w_{2\text{i}1}\right|,\ldots ,\left|w_{2id}\right|\right)}^{-1}w_{2i}^{\text{T}}, \end{array}$$

and **w**_2*i*_=[**w**_2*i*1_,…, **w**_2*id*_] is the *i*-th row of *W*_2_. For the gradient function of *g*_22_(*W*_2_) regarding *W*_2_, we decompose the problem to the gradients of *g*_22_ with regard to different rows of *W*_2_, since in the problem, the rows are independent to each other:

(27) $$\begin{array}{c} \nabla g_{22}\left(W_{2}\right)=\left[\begin{array}{c} \nabla g_{22}\left(w_{21}\right)\\ \vdots \\ \nabla g_{22}\left(w_{2m}\right) \end{array}\right], \end{array}$$

where ∇*g*_22_(**w**_2*i*_) is the gradient of *g*_22_ regarding **w**_2*i*_, and according to (25) and (26), we have the subgradient of *g*_22_ as follows:

(28) $$\begin{array}{c} \nabla g_{22}\left(w_{2i}\right)=C_{3}w_{2i}\;\mathrm{diag}{\left(\left|w_{2i1}\right|,\ldots ,\left|w_{2id}\right|\right)}^{-1}. \end{array}$$

#### 2.2.3. Updating Φ

To optimize the filters of the deep network, we fix both *W*_1_ and *W*_2_ and use the back-propagation algorithm based on the chain rule. The corresponding problem is given as follows:

(29) $$\begin{array}{c} \underset{\Phi }{\min}\left\{g_{3}\left(\Phi \right)=\frac{1}{2}{\left\|\Phi \right\|}_{2}^{2}+\overset{n}{\underset{i=1}{\sum }}\left(\frac{C_{1}}{2}{\left\|y_{i}-\left(W_{1}\phi \left(x_{i}\right)+W_{2}\phi \left(x_{i}\right)\right)\right\|}_{2}^{2}+\frac{C_{4}}{2}{\left\|W_{1}\phi \left(x_{i}\right)-W_{2}\phi \left(x_{i}\right)\right\|}_{2}^{2}\right)\right\}=\frac{1}{2}{\left\|\Phi \right\|}_{2}^{2}+\overset{n}{\underset{i=1}{\sum }}g_{3i}\left(\Phi \right), \end{array}$$

where

(30) $$\begin{array}{c} g_{3i}\left(\Phi \right)=\frac{C_{1}}{2}{\left\|y_{i}-\left(W_{1}\phi \left(x_{i}\right)+W_{2}\phi \left(x_{i}\right)\right)\right\|}_{2}^{2}+\frac{C_{4}}{2}\left\|W_{1}\phi \left(x_{i}\right)\right.-W_{2}\phi {\left.\left(x_{i}\right)\right\|}_{2}^{2}. \end{array}$$

is a data pointwise term. Back propagation is based on gradient descent algorithm:

(31) $$\begin{array}{c} \Phi ⟵\Phi -\varsigma \nabla g_{3}\left(\Phi \right). \end{array}$$

and according to the chain rule,

(32) $$\begin{array}{c} \nabla g_{3}\left(\Phi \right)=\Phi +\overset{n}{\underset{i=1}{\sum }}\nabla g_{3i}\left(\Phi \right), \end{array}$$

where

(33) $$\begin{array}{c} \nabla g_{3i}\left(\Phi \right)=\nabla g_{3i}\left(\phi \left(x_{i}\right)\right)\nabla_{\Phi }\phi \left(x_{i}\right),\\ \nabla g_{3i}\left(\phi \left(x_{i}\right)\right)=-C_{1}{\left(W_{1}+W_{2}\right)}^{\text{T}}\left(y_{i}-\left(W_{1}\phi \left(x_{i}\right)+W_{2}\phi \left(x_{i}\right)\right)\right)+C_{4}{\left(W_{1}-W_{2}\right)}^{\text{T}}\left(W_{1}\phi \left(x_{i}\right)-W_{2}\phi \left(x_{i}\right)\right). \end{array}$$

## 3. Experiments

In this section, we test the proposed method over several multitask learning problems and compare it to the state-of-the-art deep learning methods for the multitask learning problem.

### 3.1. Experiment Setting

We test the proposed method over the following benchmark datasets:

- *Large-scale CelebFaces Attributes (CelebA) Dataset*. The first dataset we used is a face image dataset, named CelebA Dataset [37]. This dataset has 2,02,599 images, and each image has 40 binary attributes, such as wearing eyeglasses, wearing hats, having a pointy nose, smiling, etc. The prediction of each attribute is treated as a task; thus, this is 40-task multitask learning problem. The input data is image pixels. The downloading URL for this dataset is at http://mmlab.ie.cuhk.edu.hk/projects/CelebA.html.
- *Annotated Corpus for Named Entity Recognition*. The second dataset we used is a dataset for named entity recognition. It contains 47,959 sentences, which contain 10,48,576 words. Each word is tagged by a named entity type, such as Geographical Entity, Organization, Person, etc., or a nonnamed entity. Moreover, each work is also tagged by a part-of-speech (POS) type, such as noun, pronoun, adjective, determiner, verb, adverb, etc. Meanwhile, we also have the labels of noun chunk. We have three tasks for each work, named entity recognition (NER), POS labeling, and noun clunking. For each word, we use a window of size 7 to extract the context, and the embedding vectors of the words in the window are used as the input. This dataset can be downloaded from https://www.kaggle.com/abhinavwalia95/entity-annotated-corpus.
- *Economics*. The third dataset we used is a dataset for tasks of property price trend and stock price trend prediction. The input data is the wave of historical data of property prices and stock prices and each data point is the data of three months of both prices of properties and stocks, and the label of each data point is the trend of stock price and property price. We collect the data of last 20 years of USA and China and generate a total number of 480 data points.

In the experiments, we split an entire dataset to a training set and a test set of the equal sizes. The training set is used to learn the parameters of the deep network, and then we use the test set to evaluate the performance of the proposed learning method. To measure the performance, we use the average accuracy for different tasks.

### 3.2. Experiment Results

#### 3.2.1. Comparison of Prediction Accuracy of Different Methods

**Figure 2:**
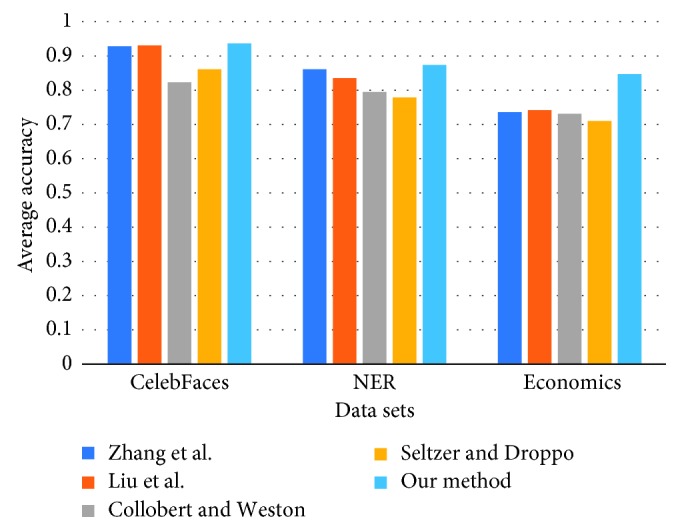
Prediction performance of compared methods over benchmark datasets.

We compare the proposed method against several deep learning-based multitask methods, including the methods proposed by Zhang et al. [33], Liu et al. [34], Collobert and Weston [15], and Seltzer and Droppo [35]. The results are reported in Figure 2. According to the results, the proposed methods always achieve the best prediction performances, over three multitask learning tasks, especially in the NER and Economics. For the Economics benchmark dataset, our method is the only method which obtains an average prediction accuracy higher than 0.80, while the other methods only obtain accuracies lower than 0.75. This is not surprising since our method has the ability to explore the inner relation between different tasks by the low-rank regularization of the weights of the CNN model for different tasks. In the Economics benchmark dataset, the number of training examples is small; thus, it is even more necessary to borrow the data representation of different tasks. For the CelebFaces dataset, the improvement of the proposed method over the other methods is slight. Moreover, we also observe that the methods of Zhang et al. [33] and Liu et al. [34] outperforms the methods of Collobert and Weston [15] and Seltzer and Droppo [35] in most cases.

#### 3.2.2. Comparison of Running Time of Different Methods

**Figure 3:**
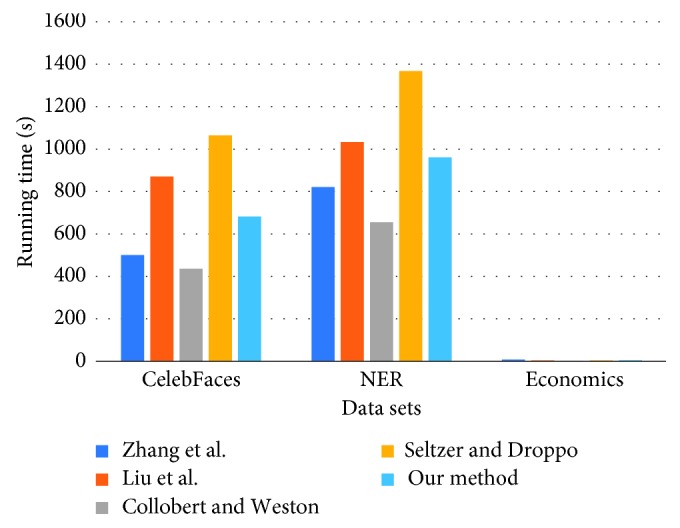
Running time of compared methods over benchmark datasets.

We also report the running time of the training processes of the compared methods in Figure 3. According to the results reported in the figure, the training process of Seltzer and Droppo's [35] method is the longest, and the most efficient method is Collobert and Weston's [15] algorithm. Our method's running time of the training process is longer than Zhang et al.'s [33] and Collobert and Weston's [15] methods, but still acceptable for the datasets of CelebFaces and NER. While for the training process over the Economics benchmark dataset, the running time is very short compared to the other two datasets, since its size is relatively small.

#### 3.2.3. Influence of Tradeoff Parameters

**Figure 4:**
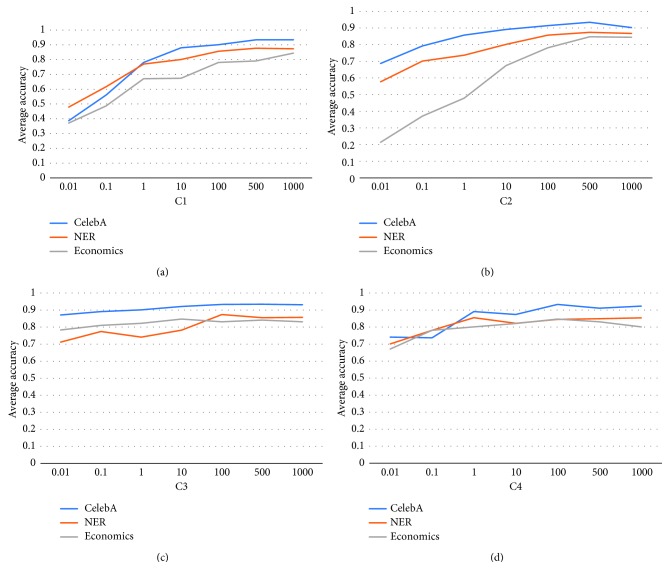
Influences of tradeoff parameters over benchmark dataset.

In our method, there are four important tradeoff parameters, which control the weights of the terms of classification errors, the rank of the weight matrix, and the *ℓ*_1_ norm sparsity of the weight matrix, and the consistency of predictions of the sparse model and low-rank model. The four tradeoff parameters are *C*_1_, *C*_2_, *C*_3_, and *C*_4_. We study the influences of the changes of their values to the prediction accuracy and report the results of our method with varying values of these parameters in Figure 4. We have the following observations as follows:

- According to the results in Figure 4, when the values of *C*_1_ increase from 0.01 to 100, the prediction accuracy keeps growing. This is due to the fact that this parameter is the weight of the classification error term, and when its value is increasing, the classification error over the training set plays a more and more important role in the learning process; thus, it boosts the classification performance accordingly. But when its value is larger than 100, the performance improvement is not significant anymore.
- When the values of *C*_2_ increases, the performance of the proposed keeps improving. This is due to the importance of the low-rank regularization of the proposed method. *C*_2_ controls the weight of the low-rank regularization term, and it is the key to explore the relationships among different tasks of multitask problem. This is even more obvious for the Economics dataset, where the data size is small, and cross-task information plays a more important role.
- The proposed algorithm seems stable to the changes in the values of *C*_3_, which is the weight of the sparsity term of the objective. This term plays the role of feature selection over the convolutional representation of the input data. The stability over the changes of *C*_3_ implies that the convolutional features extracted by our model already give good performances; thus, the feature selection does not significantly improve the performances.
- For the parameter *C*_4_, the average accuracy improves slightly when its value increases until it reaches 100; then the performances seem to decrease slightly. This suggests that the consistency between sparsity and low-rank somehow improves the performance, but it does not always help. For forcing the consistency with a large weight for the consistency term, the performance will not be improved.

## 4. Conclusion

In this paper, we proposed a novel deep learning method for the multitask learning problem. The proposed deep network has convolutional, max-pooling, and fully connected layers. The parameters of the network are regularized by low-rank to explore the relationships among different tasks. Meanwhile, it also has the function of deep feature selection by imposing sparsity regularization. The learning of the parameters are modeled as a joint minimization problem and solved by an iterative algorithm. The experiments over the benchmark datasets show its advantage over the state-of-the-art deep learning-based multitask models.

## Data Availability

The large-scale CelebFaces Attributes (CelebA) dataset used to support the findings of this study have been deposited in the CelebA repository at http://mmlab.ie.cuhk.edu.hk/projects/CelebA.html. The Annotated Corpus for Named Entity Recognition data used to support the findings of this study have been deposited in the entity-annotated-corpus repository at https://www.kaggle.com/abhinavwalia95/entity-annotated-corpus.

## References

[1] Argyriou A., Evgeniou T., Pontil M. (2007). Multi-task feature learning. *Advances in Neural Information Processing Systems*.

[2] Caruana R. (1997). Multitask learning. *Machine learning*.

[3] Doulamis N., Voulodimos A. Fast-mdl: fast adaptive supervised training of multi-layered deep learning models for consistent object tracking and classification.

[4] Evgeniou T., Pontil M. Regularized multi–task learning.

[5] Jacob L., Vert J. p., Bach F. R. (2009). Clustered multi-task learning: a convex formulation. *Advances in Neural Information Processing Systems*.

[6] Mao W., Mu X., Zheng Y., Yan G. (2014). Leave-one-out cross-validation-based model selection for multi-input multi-output support vector machine. *Neural Computing and Applications*.

[7] Ruder S. (2017). An overview of multi-task learning in deep neural networks. https://arxiv.org/abs/1706.05098.

[8] Xue Y., Liao X., Carin L., Krishnapuram B. (2007). Multi-task learning for classification with dirichlet process priors. *Journal of Machine Learning Research*.

[9] Zhou D., Wang J., Jiang B., Li Y. (2018). Multiple-relations-constrained image classification with limited training samples via pareto optimization. *Neural Computing and Applications*.

[10] Cho S.-Y., Wong J.-J. (2008). Human face recognition by adaptive processing of tree structures representation. *Neural Computing and Applications*.

[11] Owusu E., Zhan Y.-Z., Mao Q.-R. (2014). An svm–adaboost-based face detection system. *Journal of Experimental and Theoretical Artificial Intelligence*.

[12] Wong J.-J., Cho S.-Y. (2010). A face emotion tree structure representation with probabilistic recursive neural network modeling. *Neural Computing and Applications*.

[13] Yao S., Chen Z., Jia Y., Liu C. (2018). Cascade heterogeneous face sketch-photo synthesis via dual-scale markov network. *Journal of Experimental and Theoretical Artificial Intelligence*.

[14] Zhong Y., Sullivan J., Li H. Face attribute prediction using off-the-shelf cnn features.

[15] Collobert R., Weston J. A unified architecture for natural language processing: deep neural networks with multitask learning.

[16] Herath S., Ikeda T., Ishizaki S., Anzai Y., Aiso H. (1992). Analysis system for sinhalese unit structure. *Journal of Experimental and Theoretical Artificial Intelligence*.

[17] Jabbar A., Iqbal S., Akhunzada A., Abbas Q. (2018). An improved Urdu stemming algorithm for text mining based on multi-step hybrid approach. *Journal of Experimental and Theoretical Artificial Intelligence*.

[18] Lyon C., Frank R. (1997). Using single layer networks for discrete, sequential data: an example from natural language processing. *Neural Computing and Applications*.

[19] Shams R., Mercer R. E. (2016). Supervised classification of spam emails with natural language stylometry. *Neural Computing and Applications*.

[20] Geng Y., Zhang G., Li W. A novel image tag completion method based on convolutional neural transformation.

[21] Glorot X., Bordes A., Bengio Y. Domain adaptation for large-scale sentiment classification: a deep learning approach.

[22] Guo Y., Liu Y., Oerlemans A., Lao S., Wu S., Lew M. S. (2016). Deep learning for visual understanding: a review. *Neurocomputing*.

[23] Längkvist M., Karlsson L., Loutfi A. (2014). A review of unsupervised feature learning and deep learning for time-series modeling. *Pattern Recognition Letters*.

[24] LeCun Y., Bengio Y., Hinton G. (2015). Deep learning. *Nature*.

[25] Ngiam J., Khosla A., Kim M., Nam J., Lee H., Ng A. Y. Multimodal deep learning.

[26] Sadouk L., Gadi T., Essoufi E. (2018). A novel deep learning approach for recognizing stereotypical motor movements within and across subjects on the autism spectrum disorder. *Computational Intelligence and Neuroscience*.

[27] Schmidhuber J. (2015). Deep learning in neural networks: an overview. *Neural networks*.

[28] Voulodimos A., Doulamis N., Bebis G., Stathaki T. (2018). Recent developments in deep learning for engineering applications. *Computational Intelligence and Neuroscience*.

[29] Voulodimos A., Doulamis N., Doulamis A., Protopapadakis E. (2018). Deep learning for computer vision: a brief review. *Computational intelligence and neuroscience*.

[30] Wu Y., Zhai H., Li M., Cui F., Wang L., Patil N. (2017). Learning image convolutional representations and complete tags jointly. *Neural Computing and Applications*.

[31] Zhang G., Liang G., Li W. (2017). Learning convolutional ranking-score function by query preference regularization. *Lecture Notes in Computer Science*.

[32] Zhang G., Liang G., Su F., Qu F., Wang J.-Y. (2018). Cross-domain attribute representation based on convolutional neural network. *Intelligent Computing Methodologies*.

[33] Zhang Z., Luo P., Loy C. C., Tang X. Facial landmark detection by deep multi-task learning.

[34] Liu X., Gao J., He X., Deng L., Duh K., Wang Y. Y. Representation learning using multi-task deep neural networks for semantic classification and information retrieval.

[35] Seltzer M. L., Droppo J. Multi-task learning in deep neural networks for improved phoneme recognition.

[36] Zhen X., Yu M., He X., Li S. (2017). Multi-target regression via robust low-rank learning. *IEEE Transactions on Pattern Analysis and Machine Intelligence*.

[37] Liu Z., Luo P., Wang X., Tang X. Deep learning face attributes in the wild.

